# TNF-α-Induced Downregulation of ID2 in Human First Trimester Trophoblast Cells

**DOI:** 10.3923/ajbs.2025.323.332

**Published:** 2025-06-30

**Authors:** Jiayi Zhou, Yuye Wang, Meitong Chen, Yukako Kayashima, Davin Towenly-Tilson, Monawar Mohamed-Yahia Sadig, Nobuyo Maeda-Smithie, Feng Li

**Affiliations:** Department of Pathology and Laboratory Medicine, The University of North Carolina, Chapel Hill, North Carolina 27599, United States of America

**Keywords:** TNF-α, ID2, HTR8/SVneo cells, cell viability, transcription level, protein level

## Abstract

**Background and Objective:**

Low levels of Tumor Necrosis Factor-Alpha (TNF-α) support normal pregnancy, while elevated levels are linked to complications like preeclampsia by impairing trophoblast migration and invasion. The study investigates the effect of TNF-α on ID2, a protein associated with trophoblast proliferation, differentiation and stemness, using the HTR8/SVneo cell model.

**Materials and Methods:**

The HTR8 cells were treated with three different doses of TNF-α (1, 10 and 100 ng/mL) for 4 or 24 hrs. The mRNA and protein levels of ID2 were determined by qRT-PCR and western blot, respectively. Cell viability after exposure to three doses of TNF-α was assessed using the CCK-8 kit. Data are presented as Mean±SEM, analyzed using multifactorial ANOVA with *post hoc* Tukey-Kramer test in JMP 16.0 (SAS Institute, Cary, NC), considering p<0.05 as statistically significant.

**Results:**

Following exposure to three doses of TNF-α for 4 or 24 hrs, both transcriptional and protein levels of ID2 in HTR8 cells were decreased in a dose-independent manner,10 ng/mL of TNF-α had the greatest effects. Additionally, all three doses of TNF-α increased p63 expression as well. Inhibitors of NF-κB or MAPKs did not alter TNF-α-induced decrease in ID2 expression. All three doses of TNF-α increased cell viability to the same level and an inhibitor of IKK/NF-κB, BMS345541, abolished the survival-enhancing effects of the cytokine.

**Conclusion:**

The decrease in ID2 expression by TNF-α could be mediated by p63, while the increase in cell survival could be linked to NF-κB activation.

## INTRODUCTION

Preeclampsia is a hypertensive condition of pregnancy that arises after 20 weeks of gestation, marked by the development of high blood pressure along with proteinuria and potential organ damage^1^. Preeclampsia affects approximately 5–8% of all pregnancies and 75% of the cases are mild. However, if unattended, preeclampsia could progress to eclampsia, a rare life-threatening condition^2^. “Although extensively researched for decades, the exact cause of preeclampsia remains unclear. The only definitive treatment continues to be the delivery of the placenta and infant, a practice that dates back over a century”^3^.

Tumor Necrosis Factor Alpha (TNF-α), a multi-faceted cytokine, plays crucial roles in both physiological and pathological conditions related to the female reproductive system. It is essential for balancing cell fusion and apoptotic shedding of villous trophoblasts, as well as limiting trophoblast invasion into maternal decidua^4^. This limiting effect could be exacerbated by TNF-α at higher than normal levels, leading to impaired trophoblast cell invasion and sequential pregnant complications, including preeclampsia^5,6^. Impaired trophoblast cell function, resulting in insufficient maternal spiral artery remodeling and poor placentation, is considered the root cause of preeclampsia^7^. Indeed, several studies show that TNF-α suppresses trophoblast cell migration and invasion. For example, this cytokine decreases the motility of JEG cells (choriocarcinoma cytotrophoblast cell line)^8^ and HTR-8/SVneo cells (immortalized first-trimester trophoblast cell) *in vitro*^9,10^. Unlike its effects on cell motility, reports of the effects of TNF-α on trophoblast cell proliferation, growth and differentiation are inconsistent. Whiteside *et al*.^11^ reported that TNF-α induces premature trophoblast differentiation in mouse blastocyst outgrowths. Another study showed that TNF-α did not augment the proliferation of primary cytotrophoblasts, it caused growth arrest in the trophoblast cells established from first-trimester chorionic villi^12^. Additionally, TNF-α exposure increased the proliferation of JAR and JEG-3 cells^13^. In contrast, HTR8 cells showed decreased proliferation but increased cell survival after being treated with TNF-α which was reported by Silva *et al*.^14^.

Inhibitor of DNA binding protein 2 (ID2) is involved in trophoblast cell proliferation and differentiation and is considered a marker of stemness^15^. The ID2 belongs to the anti-differentiation ID protein family, characterized by structural similarity to the Basic Helix-Loop-Helix (bHLH) protein family, but without the basic domain. It acts as an inhibitor of pro-differentiation transcription elements by blocking bHLH dimerization and subsequent nuclear translocation^16^. Sustained high expression of ID2 is crucial for inhibiting trophoblast cell differentiation. Gultice *et al*.^17^ reported that the rat trophoblast cell line, Rcho-1 cells, which represent an isolated, trophoblast population committed to the giant cell lineage (equivalent to human extravillous trophoblast cell) had impaired differentiation under hypoxic conditions due to the inhibition of ID2 down-regulation. In addition, another researcher reported that ID2 overexpression prevents differentiation and ID2 knockdown results in differentiation in mouse labyrinthine placental progenitor cell line, SM10^15^.

The HTR8/SVneo (HTR8) is a well-established human first-trimester extravillous trophoblast cell line^18^. This cell line displays trophoblast progenitor cell-like characteristics, including self-renewal and expressing ID2^19–21^. The effect of TNF-α on ID2 in this type of trophoblast cells has not been reported. Accordingly, the current study examined the effects of TNF-α at three different doses on ID2 in HTR8 cells and demonstrated that the cytokine reduced expression of ID2 in HTR8 cells.

## MATERIALS AND METHODS

### Study area:

This research study was completed in 25 months. This study was carried out from May, 2022 to June, 2024.

### Cell culture:

The HTR8/SVneo (HTR8) trophoblast cell line was kindly provided by Dr. C.H. Graham, Queen’s University, Kingston, Ontario, Canada^18^ and maintained in RPMI-1640 medium supplemented with 5% FBS^22^. Cells were starved for 24 hrs without FBS, then treated with TNF-α (210-TA-020/CF, Biotech) at doses of 0, 1, 10 and 100 ng/mL for 4 or 24 hrs. Other batches of cells were treated for 24 hrs with TNF-α (10 ng/mL ), BMS (0.5, 5 and 50 μM, inhibitor of Kappa B kinase, Cayman Chemical Company)^23^, PD169316 (PD, 100 μM, Cayman Chemical Company), or SP600125 (SP, 10 μM, Cayman Chemical Company)^24^ , TNF-α (10 ng/mL)+BMS, PD or SP, cells without any treatment served as controls. At the end of the treatment, cells were collected for western blot and qRT-PCR analysis.

### Quantitative RT-PCR:

Total RNA from cells was extracted using Trizol (Life Technologies, St. Paul, MN) following the manufacturer’s instructions. The NanoDrop spectrophotometer method and gel electrophoresis were used to check the quantity and quality of RNA. The mRNA was quantified with TaqMan real-time quantitative RT-PCR (7500 real-time PCR system, Applied Biosystems, Foster, CA) by using a one-step RT-PCR kit (Bio-Rad, Hercules, CA) with GAPDH as reference gen in each reaction. The 2G^−ΔΔCt^ method was used for comparing the data^22,25,26^. The primer and probe sequences are listed in Table 1.

### Western blot (WB):

Cells were lysed in a radioimmunoprecipitation assay (RIPA) buffer and the protein concentration was determined by a BCA protein assay kit (Thermo Scientific, IL). Total protein of 20–60 μg/lane was subjected to 4–20% SDS-PAGE, then electro-transferred onto PVDF membranes. The chemiluminescence intensities of the targeted protein bands were captured using the ODYSSEY^®^ FC system and evaluated using Image Studio Software (LI-COR Biosciences, Lincoln, NE). Individual protein levels was quantified by normalizing its intensity to the β-actin in the same sample and expressed relative to the levels of the respective control group, with the mean of the control set to one. The antibodies used in the study include ID2 (MA5–32891, Invitrogen) and β-actin (#5125; Cell Signaling). Experiments were repeated three times with each experimental group consisting of three replicates.

### Cell viability assay:

The HTR8 cells were seeded in separate transparent 96-well (Falcon, 35307) plates at 1×10^4^ cells per well in 100 μL of media and allowed to attach overnight. After confluence, cells were starved with 0% FBS for approximately 18 hrs (approximately an average of 5.5×10^4^ cells). Media were discarded, cells were rinsed and replaced with 100 μL of fresh FBS-free media added with different concentrations of TNF-α or different concentrations of TNF-α plus different concentrations of BMS. After 24 hrs of treatment, CCK-8 solution (Sigma, #96992) was added to each well (final 1/10 dilution) and incubated for 1 hr. The absorbance was measured at 450 nm using a microplate reader (SpectraMax M5 Microplate Reader, Molecular Devices). The percent cell viability in individual treatment groups was determined from the optical density (OD) relative to the OD of control cells, with the control mean set to one. Experiments were repeated three times with each experimental group consisting of six to eight replicates^25,27^.

### Statistical analysis:

Data are presented as Mean±SEM. A multifactorial ANOVA test was conducted using the JMP 16.0 program (SAS Institute Inc. Cary, NC). The *post hoc* analysis was performed using the Tukey-Kramer Honest significant difference test. Differences were considered statistically significant at p-values less than 0.05.

## RESULTS AND DISCUSSION

### TNF-α increases HTR8 cell viability in a dose-independent manner:

**Fig. 1:**
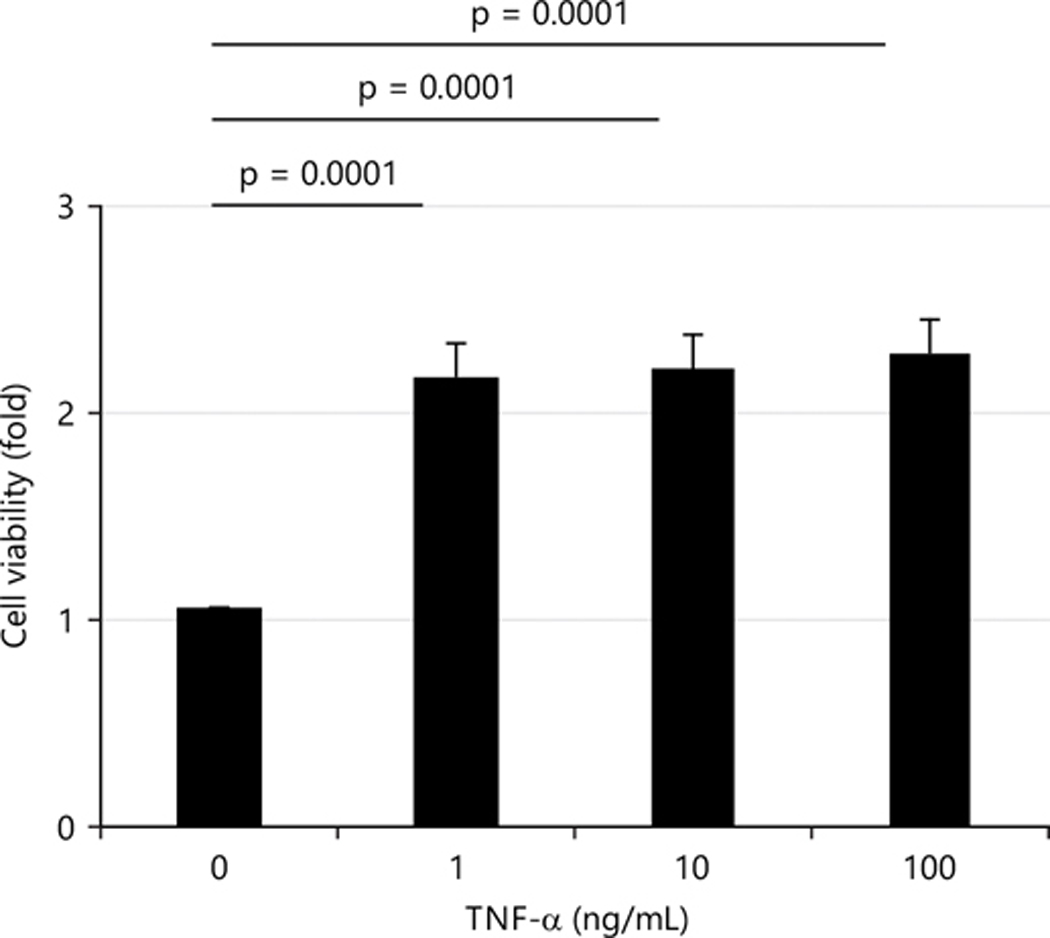
TNF-α increases cell viability in a dose-independent manner After 24 hrs treatment with three doses of TNF-α, cell viability was determined by CKK-8 kit and values are presented as Means±SEM from three independent experiments with each experimental group consisting of 6–8 replicates

Silva *et al*.^14^ reported that TNF-α (24 hrs; 10–100 ng/L) caused a small decrease (10%) in cell proliferation and an increase (9%) in cell viability. Therefore, the current study measured the cell viability after treatment with three doses of TNF-α for 24 hrs. Cell viability increased more than 2-fold by all three doses of TNF-α in this study and there were no significant differences in the effects on cell viability among the three doses (Fig. 1).

### TNF-α increases HTR8 cell viability via inhibitor of κB kinase (IKK)/NF-κB pathway:

**Fig. 2(a-b):**
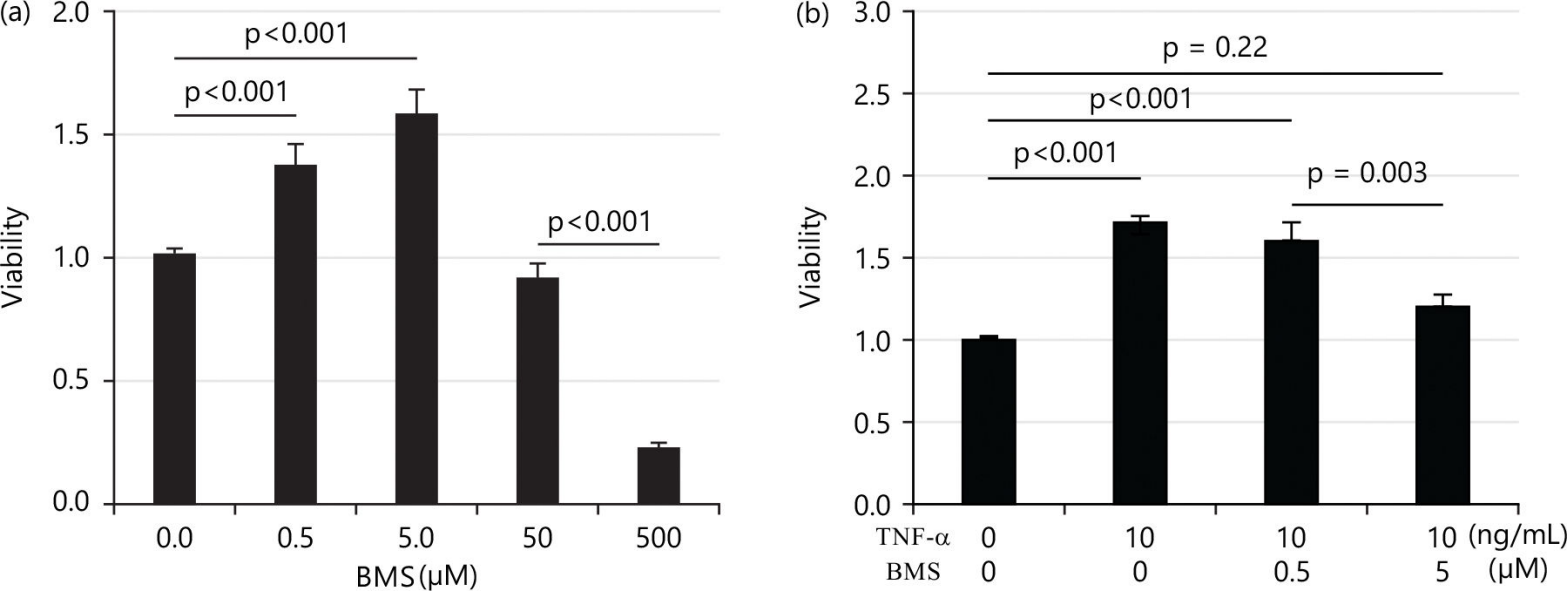
Doses of IKK (inhibitory kappa-B kinase) and inhibitors BMS345541 (BMS) in cell viability induced by TNF-α, (a) Low doses of BMS (0.5 and 5 μM) and (b) BMS in combination with 10 ng/mL Low doses of IKK (inhibitory kappa-B kinase) inhibitors BMS345541 (BMS) increase HTR8/SVneo cell viability and high dose decreases its viability while 5 μM BMS treatment abolishes the increase in cell viability induced by TNF-α, after 24 hrs treatment, low doses of BMS (0.5 and 5 μM) increased cell viability while high doses of BMS (50 and 500 μM) decreased cell viability as determined by the CKK-8 kit. After 24 hrs treatment with BMS in combination with 10 ng/mL TNF-α, only 5 μM of BMS abolished the increase in cell viability induced by TNF-α and values are resented as Means±SEM of three independent experiments with each experimental group consisting of 6–8 replicates

Studies have shown that TNF-α enhances cell survival through the NF-κB pathway^4^. To determine if the increased cell viability observed following TNF-α treatment is mediated via the NF-κB pathway, the current study used IKK (inhibitory kappa-B kinase) inhibitors BMS345541(BMS) to inhibit NF-κB activity. First, the current study tested the effects of BMS alone on cell viability. Interestingly, BMS increased cell viability at doses of 0.5 and 5 μM; but began to decrease cell viability at the dose of 50 μM and significantly decreased it at 500 μM as shown in Fig. 2a.

Therefore, the current study tested the effects of BMS on TNF-α-induced cell viability using 0.5 and 5 μM of BMS. At the dose of 0.5 μM, BMS did not alter the TNF-α-induced increase in cell viability; in contrast, at 5 μM, BMS decreased the cell viability enhanced by TNF-α as shown in Fig. 2b.

### TNF-α decreases ID2 expression in HTR8 cells:

**Fig. 3(a-b):**
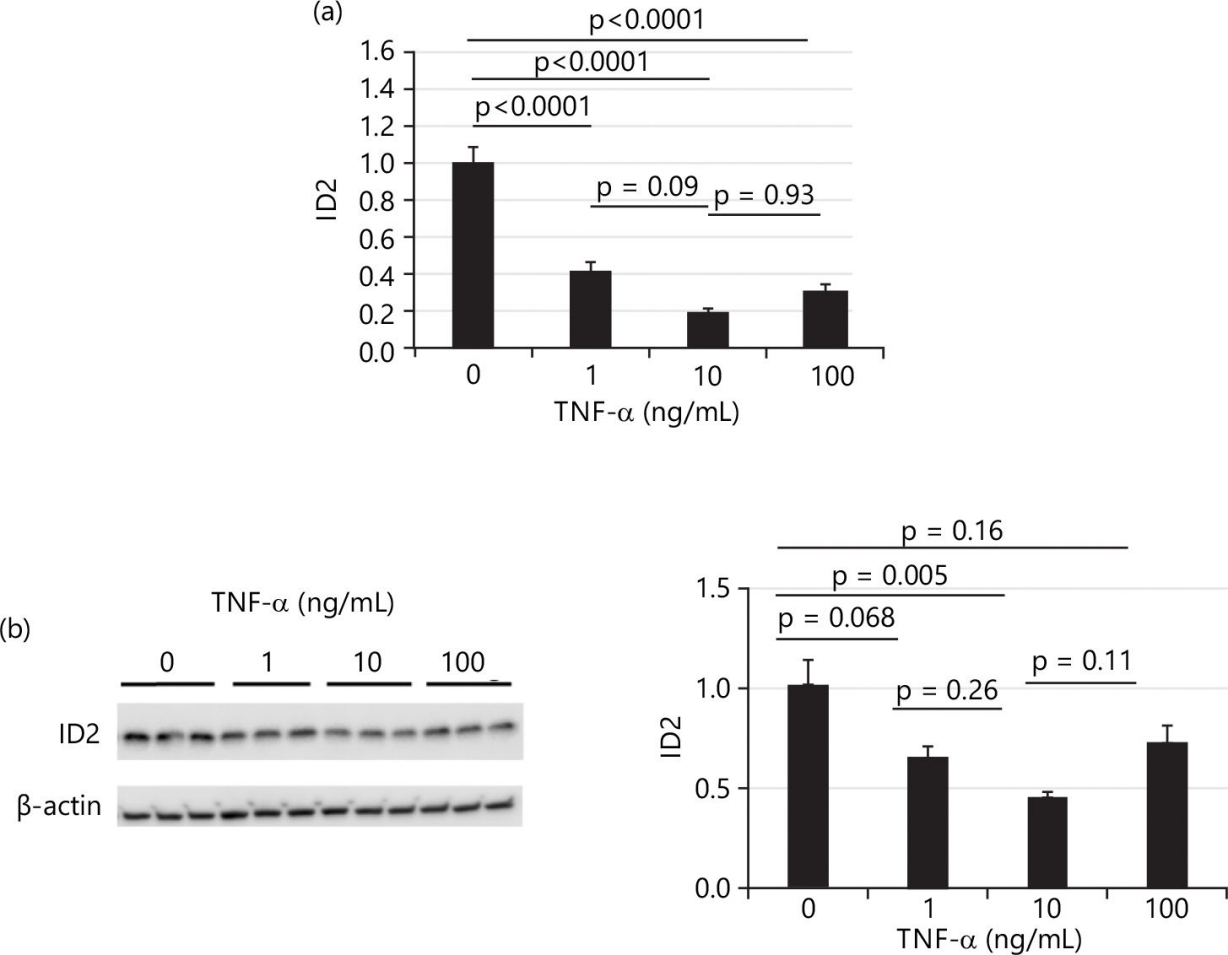
After 4 hrs treatment, TNF-α effects on transcriptional and protein levels of inhibitor of DNA binding protein 2 (ID2) in HTR8/SVneo cells, (a) mRNA levels of ID2 determined by qTR-PCR after 4 hrs of treatment with TNF-α and (b) Treatment with TNF-α, protein levels of ID2 after 4 hrs of treatment measured by western blot (left panel) and densitometric quantification (right panel) After 4 hrs treatment, TNF-α decreases both transcriptional and protein levels of inhibitor of DNA binding protein2 (ID2) in HTR8/SVneo cells, mRNA levels of ID2 were determined by qTR-PCR after 4 hrs of treatment with TNF-α. ID2 mRNA levels were significantly decreased in all three doses of TNF-α treatments. After 4 hrs of treatment with TNF-α, protein levels of ID2 were measured by Western blot (left panel) and densitometric quantification (right panel). Only 10 ng/mL dose significantly decreased ID2 protein level and values are presented as Means±SEM of three independent experiments with each experimental group consisting of 3 replicates

After 4 hrs treatment, Fig. 3a shows that the mRNA levels of ID2 in HTR8 cells were decreased by all three doses of TNF-α (1, 10 and 100 ng/mL) and the protein levels of ID2 were decreased by all three doses of TNF-α as well. Surprisingly, the cells treated with a medium dose (10 ng/mL) showed the lowest level of ID2, approximately reduced to 0.4× of control levels as shown in Fig. 3b. Meanwhile, Fig. 3b also shows that the low and high doses of TNF-α decreased ID2 protein levels to approximately 0.64× or 0.71× of control levels, respectively, although these changes did not reach statistical significance.

**Fig. 4(a-b):**
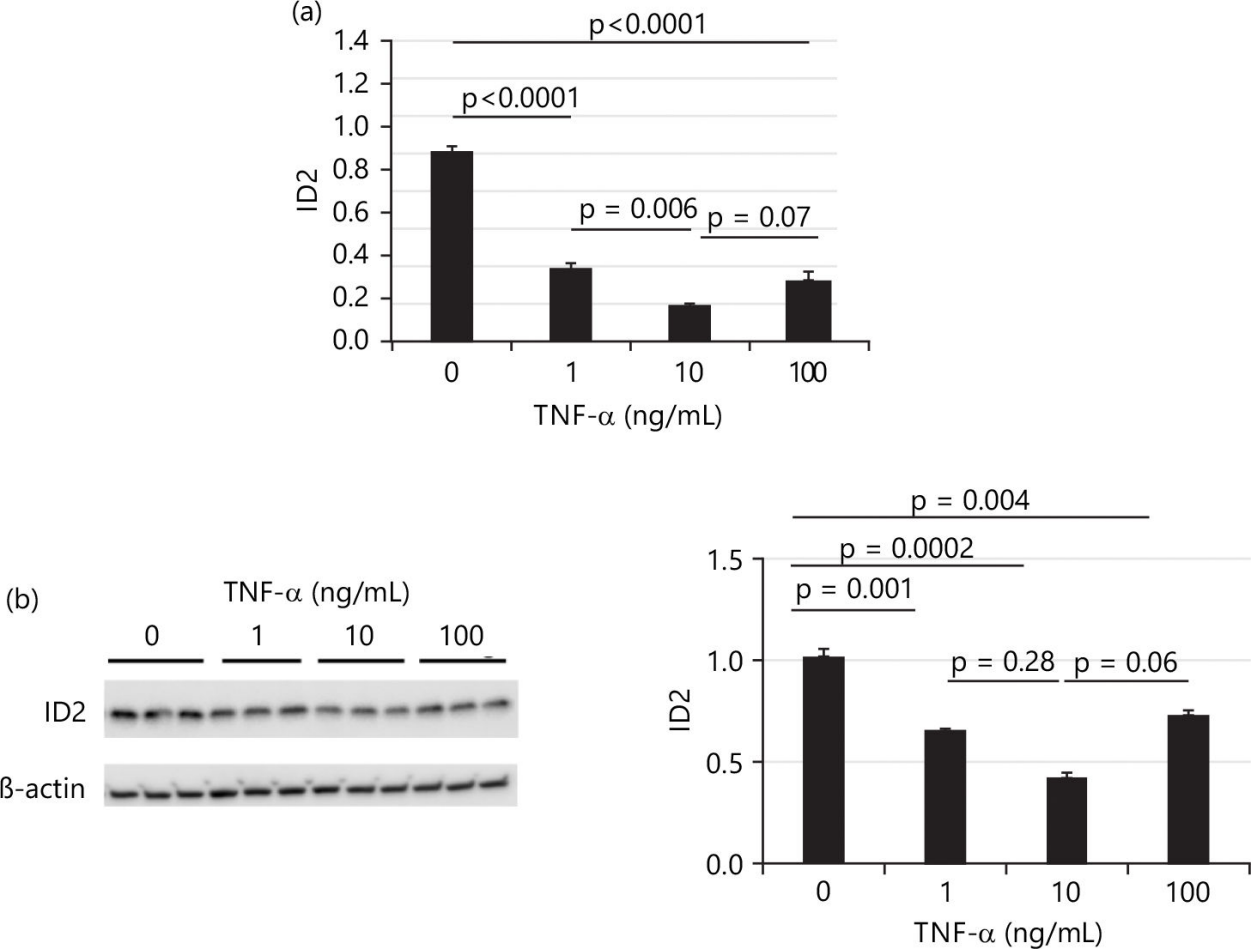
After 24 hrs treatment, TNF-α effect on transcriptional and protein levels of inhibitor of DNA binding protein 2 (ID2) in HTR8/SVneo cells, (a) mRNA levels of ID2 determined by qTR-PCR after 24 hrs of treatment with TNF-α and (b) After 24 hrs of treatment with TNF-α, protein levels of ID2 were measured by western blot (left panel) and densitometric quantification (right panel) After 24 hrs treatment, TNF-α decreases both transcriptional and protein levels of inhibitor of DNA binding protein 2 (ID2) in HTR8/SVneo cells, mRNA levels of ID2 were determined by qTR-PCR after 24 hrs of treatment with TNF-α. ID2 mRNA levels were significantly decreased in all three doses of TNF-α treatments, After 24 hrs of treatment with TNF-α, protein levels of ID2 were measured by western blot (left panel) and densitometric quantification (right panel). All three doses of TNF-α significantly decreased ID2 protein levels and values are presented as Means±SEM of three independent experiments with each experimental group consisting of 3 replicates

When treatment was extended to 24 hrs, all three doses of TNF-α significantly decreased mRNA levels of ID2. Consistent with 4 hrs treatment results, Fig. 4a shows that the medium dose of TNF-α (10 ng/mL) had the greatest effect on decreasing the mRNA levels of ID2, while the effects of low and high doses of TNF-α were comparable. Similarly, all three doses of TNF-α significantly decreased ID2 protein levels, with the medium dose showing the greatest effect (~0.6×), while the effects of low and high doses of TNF-α were comparable as shown in Fig. 4b.

### TNF-α markedly increases p63 expression in HTR8 cells:

**Fig. 5:**
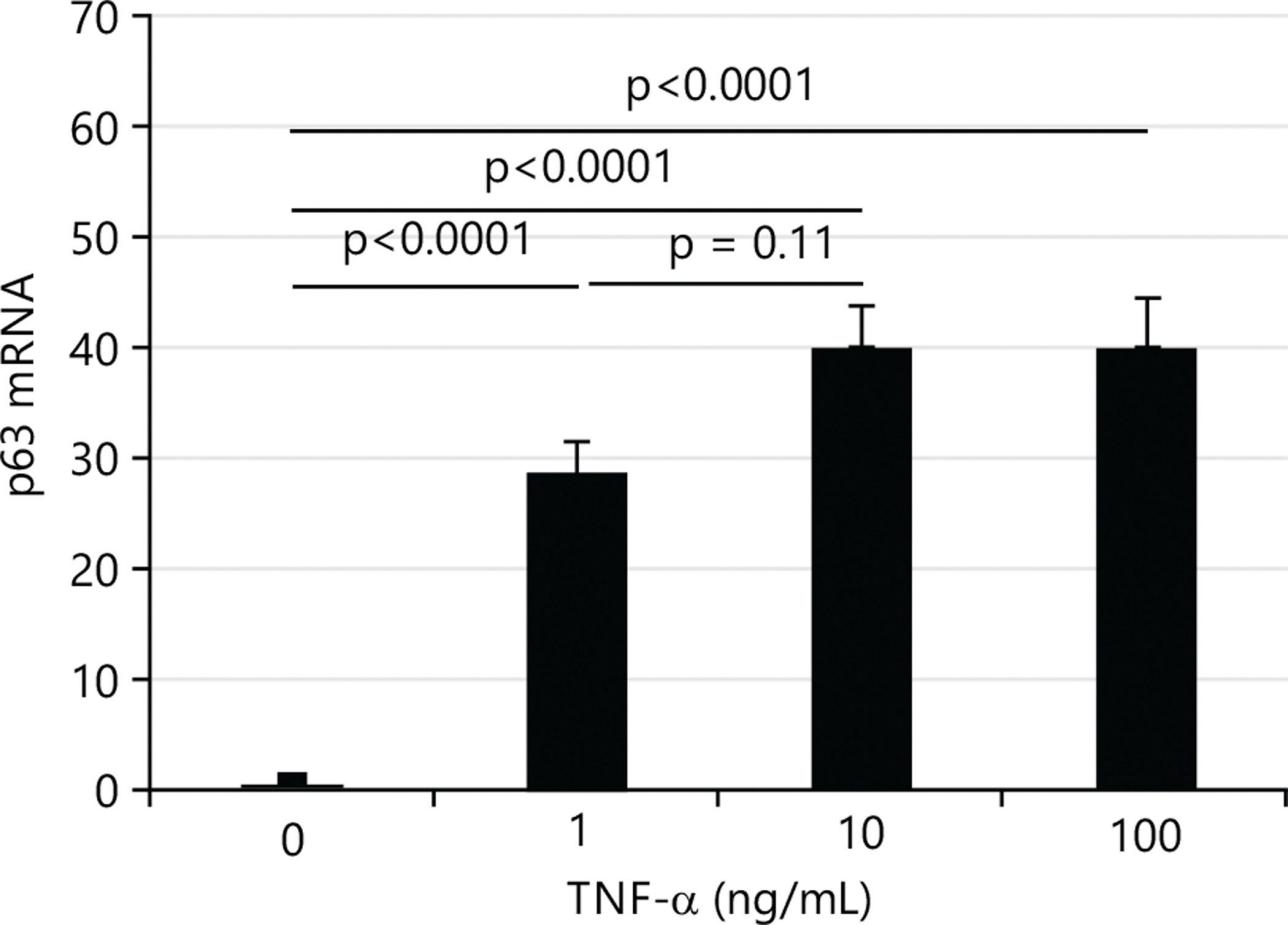
TNF-α increases the effect on the transcriptional levels of p63 in immortalized human first-trimester trophoblast cells (HTR8/SVneo) After 24 hrs treatment with TNF-α, mRNA levels of p63 were determined by Qtr-PCR and values are presented as Means±SEM

Wu *et al*.^28^ and Si *et al*.^29^ reported that ID2 is repressed by p63 and p63 is influenced by TNF-α. Therefore, the current study measured the expression of p63 in HTR8 cells treated with TNF-α. All three doses of TNF-α dramatically increased the expression of p63 as shown in Fig. 5 (~30× for 1 ng/mL, ~40× for 10 ng/mL, ~40× for 100 ng/mL TNF-α treatment).

### IKK (inhibitory kappa-B kinase) inhibitors BMS345541(BMS) does not affect the suppressing effect of TNF-α (10 ng/mL) on mRNA levels of ID2:

**Fig. 6(a-c):**
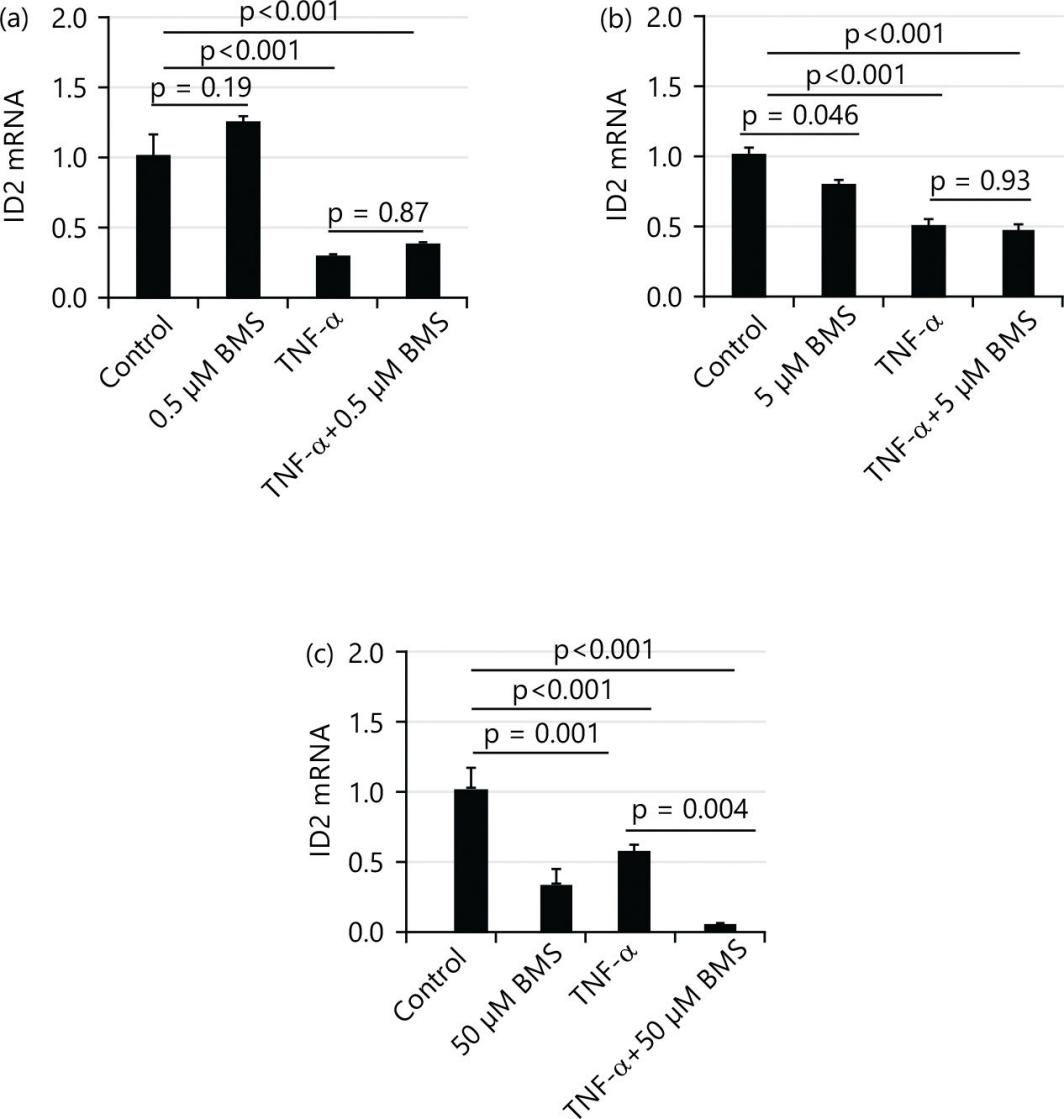
IKK (inhibitory kappa-B kinase) inhibitors BMS345541(BMS) affect the suppressing effect of TNF-α (10 ng/mL) on mRNA levels of ID2 (a) At the dose of 0.5 μM (b) At the dose of 5 μM and (c) At the dose of 50 μM At the dose of 0.5 μM, BMS alone did not affect the expression of ID2. When treated in combination with TNF-α, BMS also did not affect ID2 expression, At the dose of 5 μM, BMS alone decreases the expression of ID2. When treated in combination with TNF-α, BMS did not affect ID2 expression, At the dose of 50 μM, BMS alone decreases the expression of ID2. When treated in combination with TNF-α, BMS decreased the expression of ID2 to a greater extent and values are presented as Means±SEM of three independent experiments with each experimental group consisting of 3 replicates

To test the role of the IKK/NF-κB pathway in the effect of TNF-α on ID2, a current study used IKK inhibitors BMS345541 (BMS) to inhibit NF-κB activity. As shown in Fig. 6, at the dose of 0.5 μM, BMS did not affect the mRNA levels of ID2 while 5 and 50 μM BMS significantly decreased the mRNA levels of ID2 (~0.8× and ~0.35×, respectively). However, when treated in combination with TNF-α, only 50 μM BMS significantly decreased the mRNA level of ID2.

### PD169316 (PD, inhibitor of p38 MAPK) and SP600125 (SP, inhibitor of JNK) do not affect the suppressing effect of TNF-α (10 ng/mL) on mRNA levels of ID2:

**Fig. 7(a-b):**
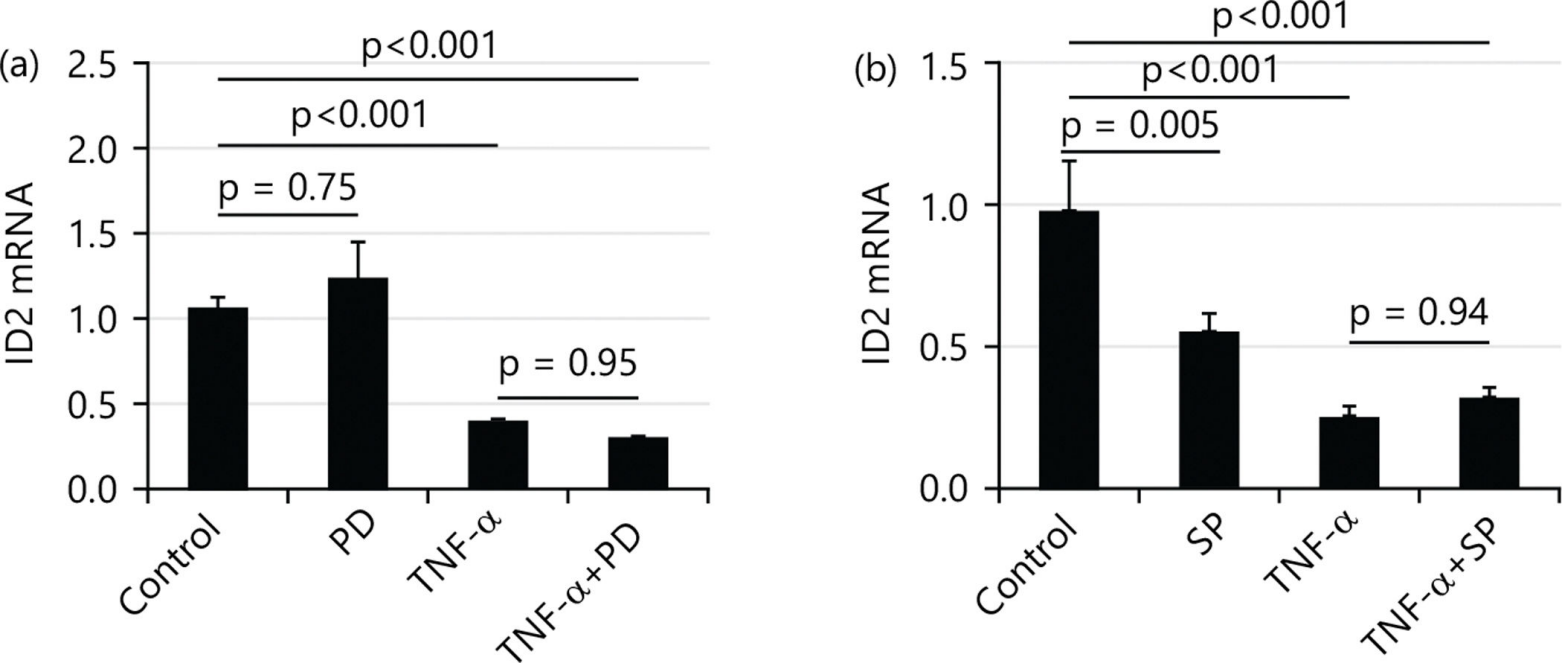
Mitogen-Activated Protein Kinase (MAPK) inhibitor, PD169316 (PD) and SP600125 (SP, inhibitor of JNK) effecton suppressing of TNF-α (10 ng/mL) on mRNA levels of ID2, (a) At the dose of 100 μM and (b) At the dose of 10 μM At the dose of 100 μM, PD alone did not affect the expression of ID2. When treated in combination with TNF-α, PD also did not affect ID2 expression, At a dose of 10 μM, SP alone decreased the expression of ID2. When treated in combination with TNF-α, SP also did not affect ID2 expression and values are presented as Means±SEM of three independent experiments with each experimental group consisting of 3 replicates

Besides the IKK/NF-κB pathway, TNF-α also executes its function through Mitogen-Activated Protein Kinases (MAPKs). The current study tested the effects of p38 MAPK inhibitors (PD169316) and c-Jun N-terminal kinase (JNK) inhibitors (SP600125) on the mRNA levels of ID2. Figure 7 shows that no reagent affected the TNF-α-induced suppression of ID2 mRNA levels. At a dose of 100 μM, PD alone did not affect the expression of ID2. When treated in combination with TNF-α, PD also did not affect ID2 expression Fig.7a.

At a dose of 10 μM, SP alone decreased the expression of ID2. When treated in combination with TNF-α, SP also did not affect ID2 expression Fig. 7b.

Current study demonstrated that TNF-α treatment decreases the expression of ID2 in HTR8 trophoblast cells, accompanied with increased p63 expression. Interestingly, among the three doses current study tested, the medium dose (10 ng/mL) of TNF-α has the greatest effects on the expression of ID2, while the low dose (1 ng/mL) and high dose (100 ng/mL) of TNF-α have the comparable effects on decreasing the expression of ID2. In addition, all three doses of TNF-α increase cell viability to the same level which could be mediated by NF-κB pathway.

Impaired trophoblast cells play an important role in pregnancy complications, including preeclampsia. The TNF-α has detrimental effects on trophoblast cells such as inhibiting their migration and invasion resulting in impaired placentation. However, the effects of TNF-α on trophoblast cell proliferation and survival remain inconclusive. Current study utilized the immortalized human first-trimester trophoblast cell line to study the effects of TNF-α as this cell line exhibits the epithelial phenotype and the proliferative and invasive characteristics of extra villous trophoblasts (EVTs)^14,18^. Here, current study show that TNF-α decreases both transcriptional and protein levels of ID2. In general, ID2 is responsible for regulating the balance between cell proliferation and differentiation. High ID2 expression is associated with proliferation while low ID2 expression is associated with differentiation. Silva *et al*.^14^ demonstrated that TNF-α exhibits an antiproliferative effect, a phenomenon observed in various trophoblastic cell types, including the ED27 first-trimester trophoblast cell line and an immortalized trophoblast cell line derived from primary trophoblast cultures. However, this effect was not consistent across all cell types, as primary cultured cytotrophoblast cells did not exhibit the same response, even when exposed to significantly higher concentrations of TNF-α^12,30^. The findings of this study suggest that the observed reduction in ID2 expression induced by TNF-α could be a mechanism contributing to its antiproliferative action in the trophoblast cells examined.

Babcock *et al*.^31^ reported that AP-1 could be a potential mechanism involved in the transcriptional regulation of ID2. In addition, AP-1 is modulated by TNF-α through p63^29^, and p63 represses ID2^28^. In the current study, TNF-α decreases ID2 expression while increasing p63 expression. The current study also found that inhibiting NF-κB and MAPKs pathways did not affect TNF-α-induced suppression of ID2 expression. Collectively, this study data suggests that TNF-α decreases ID2 expression through p63/AP-1 pathway rather than through the NF-κB or MAPK pathways.

The positive effect on cell viability reported by Stubert *et al*.^32^ was also verified in another trophoblastic cell line (BeWo cells). Current study showed that TNF-α increased cell viability in a dose-independent manner and inhibition of NF-κB pathway abolished this increasing effect of TNF-α, suggesting TNF-α increases cell viability is mediated via the NF-κB pathway.

## CONCLUSION

The study concludes that TNF-α reduces ID2 expression in HTR8 cells in a dose-independent manner, potentially mediated by p63. The TNF-α decreases ID2 expression could be via p63 and increases cell survival through the NF-κB pathway. Additionally, TNF-α enhances cell survival via NF-κB activation, as the survival-promoting effect was abolished by the NF-κB inhibitor BMS345541. Future studies should investigate the precise molecular mechanisms linking TNF-α, p63 and NF-κB in regulating ID2 expression and cell survival. Additionally, exploring TNF-α effects in different cell types or *in vivo* models could provide broader insights.

## Figures and Tables

**Table 1:** Primers and probes for qRT-PCR

Gene	Type	Sequence (5’−3’)

ID2	Hs04187239_m1	(Applied biosystems)
P63	Hs00978340_m1	(Applied biosystems)
GAPDH	Forward	GAAGGTGAAGGTCGGAGTC
	Reverse	GAAGATGGTGATGGGATTTC
	Probe	FAM-CAAGCTTCCCGTTCTCAGCC-TAMRA

## SIGNIFICANCE STATEMENT

This study highlights the regulatory role of TNF-α in modulating ID2 expression and cell survival in HTR8 cells. By identifying p63 as a potential mediator and confirming the involvement of the NF-κB pathway, these findings contribute to the understanding of TNF-α complex signaling mechanisms. The insights into TNF-α dual role in altering gene expression and promoting survival provide a foundation for exploring therapeutic targets in inflammatory or pathological conditions, particularly in placental biology or related diseases.
